# Modulating Inflammation-Mediated Diseases via Natural Phenolic Compounds Loaded in Nanocarrier Systems

**DOI:** 10.3390/pharmaceutics15020699

**Published:** 2023-02-19

**Authors:** Tojofaniry Fabien Rakotondrabe, Min-Xia Fan, Felix Wambua Muema, Ming-Quan Guo

**Affiliations:** 1 CAS Key Laboratory of Plant Germplasm Enhancement and Specialty Agriculture, Wuhan Botanical Garden, Chinese Academy of Sciences, Wuhan 430074, China; 2 Sino-Africa Joint Research Center, Chinese Academy of Sciences, Wuhan 430074, China; 3 Innovation Academy for Drug Discovery and Development, Chinese Academy of Sciences, Shanghai 201203, China; 4 University of Chinese Academy of Sciences, Beijing 100049, China

**Keywords:** polyphenolic, nanocarriers, drug delivery, bioavailability, targets, anti-inflammatory

## Abstract

The global increase and prevalence of inflammatory-mediated diseases have been a great menace to human welfare. Several works have demonstrated the anti-inflammatory potentials of natural polyphenolic compounds, including flavonoid derivatives (EGCG, rutin, apigenin, naringenin) and phenolic acids (GA, CA, etc.), among others (resveratrol, curcumin, etc.). In order to improve the stability and bioavailability of these natural polyphenolic compounds, their recent loading applications in both organic (liposomes, micelles, dendrimers, etc.) and inorganic (mesoporous silica, heavy metals, etc.) nanocarrier technologies are being employed. A great number of studies have highlighted that, apart from improving their stability and bioavailability, nanocarrier systems also enhance their target delivery, while reducing drug toxicity and adverse effects. This review article, therefore, covers the recent advances in the drug delivery of anti-inflammatory agents loaded with natural polyphenolics by the application of both organic and inorganic nanocarriers. Even though nanocarrier technology offers a variety of possible anti-inflammatory advantages to naturally occurring polyphenols, the complexes’ inherent properties and mechanisms of action have not yet been fully investigated. Thus, expanding the quest on novel natural polyphenolic-loaded delivery systems, together with the optimization of complexes’ activity toward inflammation, will be a new direction of future efforts.

## 1. Introduction

Inflammation is the stereotypical organism response actuated by the immune system to encounter and adapt deleterious tissue homeostasis caused by tissue injury, infections, or chemical agents [1,2]. Typically, inflammation acts for two main finalities, either it can be a defensive-adaptive reaction that re-establishes the organism’s homeostasis or it can be harmful when it last and pertains to destabilizing the physiological circumstances within the organism [3]. Based on the foreign stimulus and the tissue response efficiency, the inflammation can be classified as acute or chronic. Acute inflammation occurs during a short period, generally within a few weeks, until tissues complete the healing process and reach their equilibrium [4]. On the other hand, chronic inflammation lasts for a longer time, ranging from months to years. This second type of inflammation arises from unsolved acute inflammation or is commonly de novo via bypassing the initial inflammation step. Recent uncoverings have attributed the important roles of the inflammatory phenomenon in the early stage of degenerative disorders pathology, which progressively turn to chronic diseases, including arthritis, neurodegenerative conditions, skin inflammations, inflammatory bowel diseases, metabolic syndromes, and cardiovascular diseases (CVD). According to the World Health Organization (WHO), these inflammatory-based chronic diseases have increased worldwide and present great menaces to human life, as they lead to high rates of mortality (≥60%) [2].

So far, a plethora of synthetic anti-inflammatory drugs, including steroids, non-steroidal anti-inflammatory drugs (NSAIDs), and glucocorticoids, have been developed and clinically used for the treatment of these diseases. However, these drugs have mostly expensive and prompt short- or long-term side effects, in addition to their efficacy [5]. A large number of scientific investigations have recently proved that natural phenolic compounds, encompassing flavonoids, phenolic acids, and stilbenoids, displayed promising effects in managing these types of inflammatory-mediated diseases. In addition to their great therapeutic potential, these compounds are inexpensive to produce and present fewer side effects and toxicity. Phenolic curative effects are explained by a variety of mechanisms, which consist mainly in silencing inflammation development. However, the physicochemical (size, structures, polarity) and poor pharmacokinetics properties (absorption, distribution, metabolism, excretion, toxicity: ADMET) of these compounds sometimes refrain from the mass production of phenolic-based marketable pharmaceuticals. Additionally, their ultimate therapeutic effects can be hampered by the low bioavailability to the organisms, as well as the abject targeting efficacy, due to instabilities and/or low final concentrations delivered to the targets [6,7].

Within this framework, drug delivery systems (DDS) have arisen during the last five decades to enhance pharmacokinetics properties, increase target site carriage, and stabilize these chemicals to improve their therapeutic activities. Among them, nanocarrier delivery systems (NDS) have recently demonstrated promising results for attaining the above-cited objectives in a more controlled manner [8]. Phenolic-loaded NDS consists of encapsulating, entrapping, or decorating phenolics with one or a variety of materials to form a final complex of the nanoscale range. Regarding the nature of the carrier materials, NDS could be classified as organic, inorganic, or hybrid. Organic NDS consists of biocompatible carbon-based vesicles, such as liposomes, solid lipids, micelle, polymeric, and hydrogel nanocarriers. However, those formed with inorganic-based elements, such as metals, constitute the second group. Hybrid NDS types are synthesized when these organic and inorganic materials are compounded to form nanoparticle complexes.

This present review paper synthesizes and discusses the recent progress of the common phenolic-loaded NDS in the management of inflammatory-based diseases. Although nanocarrier technology offers a variety of advantages in polyphenols delivery, the synthesized complexes’ mechanisms of action have not yet been systematically explored. Additionally, many of the demonstrated potential distinctive phenolic compounds research is limited to their coarse form uses. Hence, broadening the application of nanocarrier systems to a variety of bioactive phenolics, along with the optimization of complexes to inflammatory target efficiency, will be a new focus of future endeavors.

## 2. Natural Phenolic Compounds in Inflammatory Mitigation and Their Challenges

**Figure 1:**
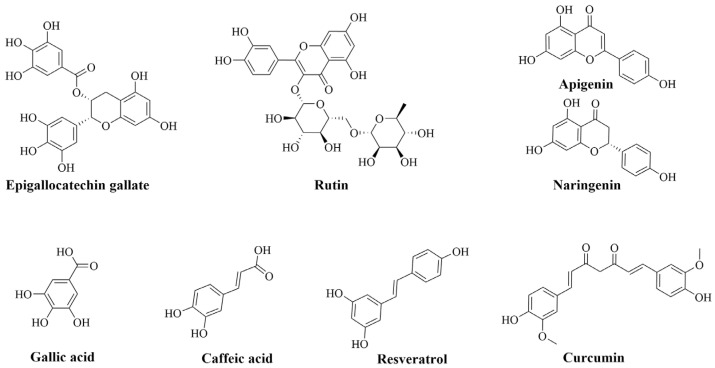
The common natural phenolic compounds studied in this review.

Flavonoids and phenolic acids are the most abundant phenolic compounds [9,10]. Amongst flavonoids, EGCG (catechin), rutin (flavonol), naringenin (flavonone), and apigenin (flavone) are the most abounding (Figure 1) [11]. However, ellagic acid and caffeic acid are among the most prevalent phenolic acids [12]. In addition to the antioxidant effects of phenolic acids to promote health improvements, their anti-inflammatory roles add more benefits for the management of different impairments. So far, much research has demonstrated the ability of these types of phenolics to mediate various types of inflammatory-based diseases. In vitro studies have demonstrated their ability to hinder the differentiation of Th1 and Th17 in the pathogenesis of rheumatoid arthritis. Additionally, they could downregulate pro-inflammatory cytokines and chemokines production via their interference with IL-1β signaling pathways or with laminin receptor (67LR) [13,14,15]. In neuroinflammation, a lot of evidence has demonstrated that flavonoids can protect neurons towards activated microglial cells, mediate the production of inflammatory markers, and regulate pro-inflammatory signaling pathways (MAPK, JAK/STAT) [16,17,18]. The beneficial intestinal inflammatory mediation of flavonoids was also evidenced. They can hinder eicosanoids (COX, LOX, and leukotrienes) synthesis and overexpression, downregulate the production and hiring of immune cells, which reduces the releases of pro-inflammatory effectors, and modulate the associated signaling pathways [19,20]. Along with the several proofs supporting the beneficial effects of flavonoids on carbohydrates and lipid homeostasis in the organism, they have been also demonstrated to reduce the production of inflammatory molecules and improve the activation of the peroxisome proliferator-activated receptor gamma (PPAR-γ) [21,22,23].

In addition to the above-mentioned phenolic classes, there are also other popular efficient phenolic compounds, such as curcumin and resveratrol. These compounds are extensively reported to express favorable pharmacological actions in managing diverse inflammatory-based diseases. Many references have defined their efficacies towards RA by decreasing the levels of inflammatory markers and the associated matrix metalloproteinases (MMP), hindering activation of cytokines produced by T cells, and regulating the balance of Th17/Treg [24,25]. Regarding their effects on neurodegenerative diseases, it has been supported that they can inhibit the overactivation of microglial cells, downregulate the neuroinflammatory markers, and help to clear the aggregated β-amyloid [26,27]. Their effects in IBD were promising and explained by their ability to obstruct neutrophils cells infiltrations, downregulate eicosanoids and iNOS overproductions, and lower T-cell inflammatory responses [28,29,30,31]. By blockading the NF-KB pathway via upregulation of SIRT-1 and AMPK functions, resveratrol can attenuate the inflammatory cascade in metabolic disorders [32].

Despite these various positive effects of phenolic compounds on human health, they have their limitations and challenges. First of all, they are easy to degrade, due to the auto-oxidation, epimerization, hydrolysis, and crystallization during the storage period or processing [33]. Various factors may affect these instabilities, involving solvent dissolution and alkalinity, the surrounding temperature and oxygen availability, and the interaction with oxygen and other molecules [34]. Secondly, their bioavailability (ADMET) to human organisms is very poor, due to their physicochemical features. We can cite their poor water dispersibility, intestinal uptakes via the transport mechanisms, and the different metabolic reaction changes within the gastrointestinal tract throughout the digestion [6,7]. As most of the promising pharmacological potentialities of these compounds have been evaluated in vitro and in vivo, their bioefficacy in real conditions should be accompanied by a delivery system that helps their release at the targeted site of action [35]. The nanocarrier system loaded with phenolic compounds is one of the most utilized strategies to overcome these challenges, which, in parallel, improves their physical appearances.

## 3. Nanocarriers Systems Used for Loading Phenolic Compounds

The recourse of nanocarrier systems in drug delivery has gained much interest since its initiation around the mid of 19th century. Physicists started to develop the conjugation of drugs to polymers in 1955, and during the first two decades, the formulation of liposomes-based drugs engaged many researchers [36]. The discovery of some liposome limitations in specific targets and the advancement of the technology led to the development of new strategies, such as the colloidal-based nanoparticles, which were rapidly approved by the U.S. Food and Drug Administration (FDA) [37,38]. Currently, a variety of nanocarrier encapsulations have been exploited, which could be classified into organic and inorganic nanocarriers. Among the organic types, we could cite solid lipid nanoparticles, lipid nanoparticles, polymeric nanoparticles, and hydrogels. However, mesoporous silica and metallic nanocarriers are frequently used as inorganic nanocarriers.

### 3.1. Organic Nanocarriers

Solid lipid nanoparticles (SLNPs) are formed of a colloidal system prepared by the dispersion of a solid lipid matrix, such as fatty acids, some glycerides, and steroids, in the presence of surfactants for stabilization and having a final size ranging from 50–10,000 nm [39]. The loaded compounds are incorporated within the solid lipid central core. This nanocarrier type is suitable for a wide range of administration routes and significantly ensures the stability of the loaded compound from degradation [40].

Liposomes are formed of a lipid-based vesicles shell made with amphiphilic molecules (phospholipids) and sometimes stabilized with niosomes, with a final size ranging from 250 nm to 2500 nm [41,42]. The features of liposomes can be handled by varying the type, amount, and size of phospholipids, as well as their mode of production. In general, producers add cholesterol to enhance their rigidity and stability [43]. The crucial advantage of liposomes concerns their capability to monitor the encapsulated drug release at the targeted site of action with higher molecule-loading ability, biodegradability, and low toxicity [44].

Polymeric-based nanocarriers, also known as sub-micron solid particles, are colloidal vesicles built with biodegradable polymers, whether natural (proteins or polysaccharides) or synthetic, with a final size of 10 nm to 1000 nm. Depending on the manufacturing techniques and the encapsulation mode, polymeric nanocarriers could be nanospheres, nanocapsules, dendrimers, or micelles [45]. The loaded drug is dispersed evenly within the polymer matrix in the nanosphere when they are entrapped in small cavities made by the polymeric membrane in nanocapsules [46]. Polymeric micelles are constituted by assembling amphiphilic copolymer structures, which can be di-block or tri-block, in which the loaded drugs are incorporated within the internal hydrophobic core of the micelles [46]. Beyond other nanocarriers, the drug release control can be monitored well with an extended circulating time in the organism when using this type of delivery system. It is also recognized to be more stable within the blood flow [41,47].

**Figure 2:**
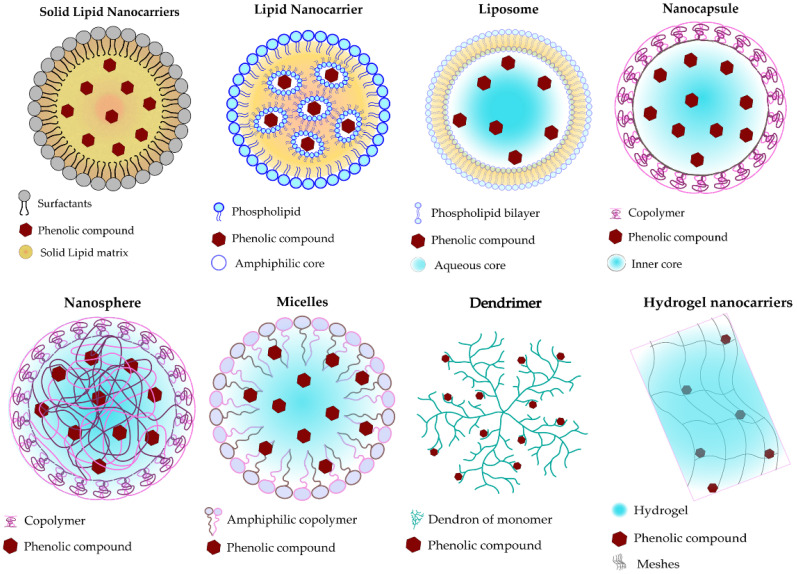
Schematic representation of organic nanocarriers used for loading polyphenolics.

Hydrogels nanocarriers consist of colloid hyper-branched polymers that are capable of absorbing large amounts of fluids and can swell in an aqueous milieu. The loaded drugs are entrapped within the mesh cross-links of the gel awaiting their release after the hydrogel reaches equilibrium and are triggered by adequate stimuli (temperature, pH, ionic strength, and light) [48]. The crucial benefits of using hydrogel are the prominent increase of the drug bioavailability, in a controlled manner, and its great variety of administration routes [40]. The schematic representation of these types of organic nanocarriers is represented in Figure 2.

### 3.2. Inorganic Nanocarriers

Mesoporous silica has received a lot of interest in drug delivery, due to its biocompatibility, high specific surface area, and porous structure, which can load an important amount of drugs [49]. All types of drugs, lipophilic or hydrophilic, can be charged within the controllable size pores (20 nm to 50 nm) [50]. The mesoporous silica is initially obtained from melting inorganic silicon dioxide (SiO_2_) powders at high temperatures before leaching out the metal oxide to obtain the porosities [51].

**Figure 3:**
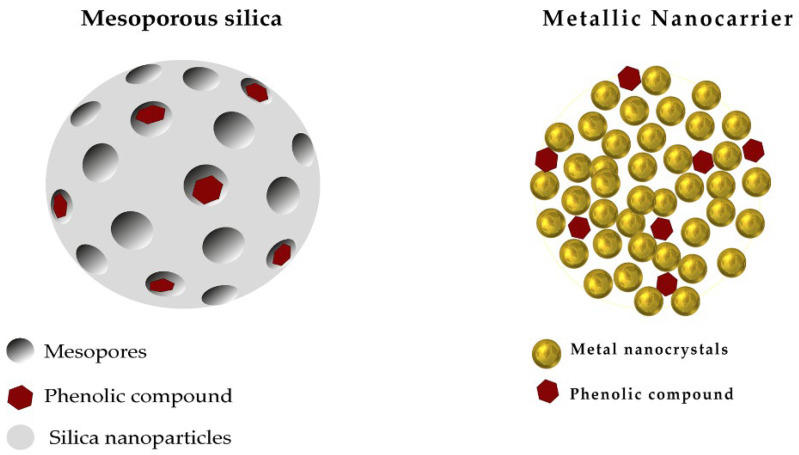
Schematic representation of inorganic nanocarriers used for loading polyphenolics.

Metallic nanoparticles are tiny particles of 1 nm to 20 nm made with stable metals, such as gold, silver, palladium, zinc, and titanium, whose surfaces are easily functionalized and facilitate the conjugation with active drugs through various interactions modes (covalent bond, hydrogen bonds, and electrostatic) (Figure 3). The drugs are then loaded at the coated surface of the metals to ensure their optimal release at the target site. Such nanoparticles could be synthesized from a bulk material size reduction approach, such as lithography and laser ablation, or via atom assembling techniques, such as flame spraying, laser pyrolysis, and microemulsion [52]. Depending on the delivery purposes, metallic nanoparticles can be coated with non-ionic or charged coatings with diverse functions (polymers, surfactants, etc.) [53,54]. The tiny size of metallic nanoparticles allows them to link smoothly with the organism’s biomolecules, which warrants the specific target area delivery and fewer side effects [55]. It is of note that the functionalization or conjugation of nanocarrier systems are made with targeting ligands, peptides, proteins, or antibodies to promote the recognition of the complex to the targeted cells receptors and achieve active targeting [56,57].

## 4. Designed Polyphenolic-Loaded Nanocarriers for Inflammation-Mediated Diseases

### 4.1. Polyphenolic Nano Delivery for Rheumatoid Arthritis

Rheumatoid arthritis is one of the inflammation-mediated diseases and is characterized by continuous inflammation of the synovial joints, and it can result in lasting sufferance, disability, and even early death [58]. The joints start swelling when the synovium is subjected to hyperplasia by an inner proliferation of macrophages and fibroblast cells. Additionally, this hypertrophied synovium invades the adjacent tissues, such as the cartilage and bone surfaces, that enhance inflammatory responses. Therefore, it has been reported that high amounts of pro-inflammatory mediators, such as TNF-α, IL-1, IL-6, and IL-17, are quantified in RA [59]. Additionally, this TNF production has been reported to be triggered by the autoantibodies ACPAs. However, these cytokines mitigate the main function of the cartilage-replenishing matrix and chondrocytes through nitric oxide (NO) stimulation, as well as other pro-inflammatory signal releases [60].

Recent investigations have improved the delivery of phenolic compounds for managing arthritis. Zheng et al. have improved the antiarthritic of EGCG by formulating optimized casein nanoparticles loaded with an EGCG glucosamine matrix (EGCG/GA/casein). This formulation dispersion was found to be highly stable, even after 12 months of storage, and showed excellent inhibitory effects on fibroblast-like synoviocytes arthritis cells in vitro. Similarly, the oral administration of EGC-NPs suppressed arthritis aggravation in collagen-induced arthritis (CIA) rats, which was explained by significant downregulation of TNF-α, IL-1β, IL-6, and IL-8 cytokines [61]. Another nanomicelle loaded with a combination of the antiarthritic drug 9-aminoacridine (9AA) and caffeic acid was also developed by conjugating caffeic acid with methoxy polyethylene glycol (mPEG) and ε-caprolactone (mPEG-β-PCL) to form 9AA-NMs. The intraperitoneal injection of 9AA-NMs reduced the severity of arthritis inflammation in CIA Wistar rats, which was due to the synergistic activities on the blockading NF-kB pathway and triggering the orphan nuclear factor NR4A1 in arthritis [62].

In terms of flavonoids, a comparison of different solid lipid-based nanocarriers (stearic acid, stearic-lauric, or lecithin-chitosan) loaded with naringenin resulted in the effective activity of this encapsulation within stearic-lauric (Nar-SL). In addition to the extended releases, Nar-SL also diminished the inflammatory factors, together with the degradation of the joint in CFA-induced RA. The complementary anti-inflammatory action between naringenin and lauric acid was supposed to be the principal reason [63]. Another study by Mohanty’s research group, in 2020, combined naringin with phenethyl isothiocyanate (PEITC) in a liposome carrier (1,2-dipalmitoyl-sn-glycero3-phosphocholine/cholesterol/1,2-stearoyl-sn-glycerol-3-phosphoethanolamine-020CN (DPPC/Chol/DSPE-020CN)). The intraperitoneal injection of this combinatorial formulation for 3 weeks increased the anti-inflammatory cytokine IL-10 level and attenuated bone erosion. The immune cell infiltrations in Freund’s complete adjuvant (FCA)-induced arthritis were also hindered. A synergistic enhancement of the antiarthritic action of naringenin and PEITC was determined from this preparation [64]. The same research group lately investigated the delivery optimization of naringin encapsulated within a biodegradable polymer PLGA (NAR-PLGA-NPs). A sustained release of the loaded compound in the intestine was discovered when stabilizing NAR-PLGA-NPs with poloxamer and sodium dioxylate. In the meantime, a significant increase of the anti-inflammatory marker IL-10, as well as the attenuation of RF and C-reactive protein (CRP) releases, were discovered in arthritic-induced rats [65]. A nanocrystal strategy through the wet milling of naringenin with a co-poloxamer (F127) was also applied and resulted in bioavailability improvement and synovial damage, as well. Oral administration of nanocrystal has increased the cellular uptake and diffusion in CIA-induced RA model [66].

Metallic nanocarriers, loaded with resveratrol, using ruthenium as the core product and coated with PLGA-dextran sulfate (QRu-PLGA-RES-DS), were developed to improve the bioefficacy of resveratrol in RA. The self-assembly of the formulation has enhanced the bioavailability and circulating time of resveratrol, while triggering macrophage polarization to the M2 phenotype. Meanwhile, M1 macrophage infiltration at the synovia was reduced, which signifies the antiarthritic strength of this elaborate core-shell structure [67]. A mixed micelle, built of a mixture of poloxamer 188 and poloxamer 407 coated with poly-lactic acid (PLA), was also designed to load resveratrol for intra-articular injection. One week of the co-micellar injection on CFA-induced RA was enough to reduce the synovium swelling, which correlated with the reduction of TNF-α levels, as well as the cartilage replenishment [68]. Combinatorial application of methotrexate and resveratrol (MTX-RSV) nanoemulsion loaded in gel (carbopol 940) showed a greater inhibition of inflammation (78.76 ± 4.16%) and anti-arthritic effects in vivo than those loaded individually [69]. Likewise, the oral administration of resveratrol loaded in hydrogel (cellulose aerogel) was demonstrated to exert a great impact on arthritis model management. In addition to the bioavailability amelioration of resveratrol, this delivery system also helped to induce the anti-inflammatory effects by downregulating the P38 pathway and activating SIRT-1 expressions [70].

**Figure 4:**
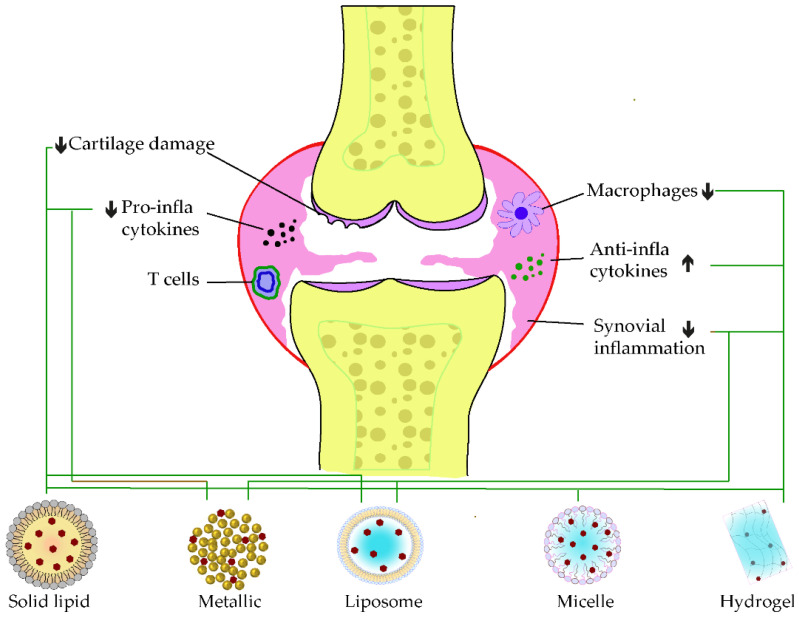
Examples of nanocarrier delivery of polyphenolics and their targets in Rheumatoid arthritis. The black arrows up signify the increasing while the down ones indicate the decreasing effects. The black dots in the figure consist of pro-inflammatory cytokines, the red dots are anti-inflammatory cytokines, and the yellow dots represent the bone marrow. The bold lines match the nanocarriers and their respective targets.

When loaded alone in solid–liquid nanocarriers, curcumin showed promising potential in ameliorating the synovial inflammation of CFA-induced models by reducing immune modulation and inflammatory cascade [71]. However, a co-encapsulated formulation of resveratrol and curcumin in lipid nanoparticles showed pronounced antioedematogenic efficiency and cartilage damage protection than the single-loaded standard in CFA-induced models. The lipid core of the product was formed of grape seed oil, and the emulsifier was sorbitan monostearate and polysorbate 80, in which an equal amount of the two phenolic standards were incorporated [72]. On the other hand, a HAS-based developed nanoparticle was recently developed to co-deliver curcumin and prednisolone. This latter exerted a more extended circulating time of curcumin before release, due to the attachment of the loaded drug in the albumin mesh. Furthermore, the synergistic anti-inflammation effect of curcumin and prednisolone was discovered with an apparent accumulation at the targeted site of action [73]. Another curcumin delivery carrier using gel nanoemulsion was developed for its anti-arthritic effect. The nanoemulsion increased the permeability of the carrier and resulted in a significant decrease of inflammatory markers, notably TNF-α and IL-1β, while its gelation within carbopol-980 enhanced the topical anti-arthritis efficacy on CFA-induced model paw [74]. The incorporation of curcumin in polymeric nanocarrier, based on carboxymethyl cellulose acetate butyrate (CUR-CMCAB) resulted in a promising release time and anti-arthritic efficiency when taken orally by CFA-induced rats. These were explained by the complete dispersal of curcumin to its amorphous form in the polymeric shell and the improvements seen from the different behavior testing along the treatment [75]. In 2018, Fan et al. entrapped curcumin in hyaluronic acid to form nanomicelle particles, in which the intraperitoneal injection significantly decreased the swelling of CFA-induced arthritis models. A reduced level of VEGF expression and inflammatory cytokines was determined following the treatment. In parallel, it improved the lubrication of joint cartilage in arthritis [76]. Kang and coworkers formulated an acid-triggered micelle particle that releases instantly the loaded curcumin once in an acidic environment. In monosodium iodoacetate (MIA)-induced osteoarthritis, the injection of this micelle solution blockaded pro-inflammatory factors expressions, such as IL-1β and TNF-β. In addition, this latter protected the degradation of the cartilage in the synovium [77]. Figure 4, below, illustrates some examples of applications of phenolic-loaded nanocarriers applied in rheumatoid arthritis.

### 4.2. Polyphenolic Nano Delivery for Neurodegenerative Disease

Neurodegenerative disease is among the crucial health-threatening issues affecting learning abilities and memory, especially for old people. It is commonly manifested in the form of Alzheimer’s and Parkinson’s diseases. In addition to the abnormal aggregation of neuronal proteins, recent findings have demonstrated that inflammation plays a critical role in the pathogenesis of this disease [78]. Alzheimer’s disease (AD) is characterized by the extraneuronal aggregations of amyloid-β (Aβ) proteins, intraneuronal formation of neurofibrillary tangles from hyperphosphorylation of tubulin-associated unit (tau) protein, and neuronal damages [79]. Parkinson’s disease (PD) mostly impacts the motor system. The hallmark of this disease consists of a midbrain gradual loss of dopaminergic neurons, accompanied by the intracellular aggregation of α-synuclein (α-syn), also known as Lewy bodies (LB) [80]. Likewise, neuro-inflammation caused by microglia plays a pivotal function in the pathogenesis and development of PD.

A great number of delivery systems loaded with phenolic compounds have been pioneered for improving their neuroprotection efficiency and target deliverance. It has been reported that nanoparticles formed with EGCG in PLVA-PEG-PLA (nanoEGCG), when administrated orally, not only improve the bioavailability of EGCG, but also mitigate the release of Aβ plaques, as well as the levels of Aβ_1–42_, in Alzheimer-induced rats. Additionally, this nanoEGCG was attested to reduce the expression of amyloid precursor proteins, acetylcholinesterase, and glycogen synthase kinase-3 beta (GSK-3β), while the levels of 3-phosphoinositide-dependent protein kinase-1 were found to be rising [81]. Another work of Cano and coworkers reported that polymeric nanocarriers built with co-entrapped EGCG and ascorbic acid within PEGlated-PLGA can smoothly pass the blood–brain barrier (BBB) and enhance the neuro-inflammatory effects of EGCG, together with synaptogenesis in APP/PS1 Alzheimer mice. Similarly, the aggregation of Aβ plaques and Aβ_1–42_ levels declined [82]. Given the effectivity of PEGlated-PLGA encapsulation, recent work has mended EGCG loading by co-encapsulation with shRNA, a β-site APP cleaving enzyme antisense (BACE1-AS), and the RGV29 peptide. This nanocarrier formulation sustained the circulation time of the loaded drugs in the bloodstream and facilitated their ability to pass across the BBB. Their neuroprotection effects at the targeted site, the brain, were promising by reducing Aβ levels and mitigating BACE1 function [83]. In PD, Li et al., 2020, formulated diverse DSPE-PEG B6-based micelles coated with EGCG (B6ME-NPs). To ease their traceability throughout the deliverance, they incorporated SPIONS in the core shell of the particle. The intravenous administration of these formulations has improved the EGCG stability and delivery to the brain by B6 contribution in overcoming the BBB and inhibited noticeably the aggregation of α-syn agglomeration in vitro [84]. Selenoprotein analogs (Tet-1) conjugation of selenium nanoparticles coated with EGCG (Tet-1-EGCG@Se) has also well-enhanced the inhibition of Aβ aggregation and fibrillation in AD. The Tet-1 functionalization was found to facilitate the cellular uptake of the formulation, while Tet-1-EGCG@Se reversed the fibrillation of Aβ agglomerate to its native form [85]. A recent investigation showed the effective action of EGCG-Se nanoparticles in neuroprotection via reducing the microglia inflammation in LPS-induced PC12 cells (in vitro) and in the targeted site of a spinal cord injury rat model (in vivo) [86].

Rutin loaded in solid lipid nanoparticles has also shown great stability and circulating time in the blood flow. In addition, this nanoparticle can well-infiltrate the BBB, which suggests its efficient brain target delivery [87]. Similarly, oral administration of phospholipidic nanocarrier, incorporated with rutin, exhibited eminent brain concentration and attenuated neurological, as well as ischemic, damage in middle cerebral artery occlusion (MCAO) rats [88]. A metallic nanocarrier loaded with rutin and coated by the traceable red congo facilitated the localization of Aβ plaques by imaging. Additionally, this theranostic nanoparticle significantly attenuated the cytotoxicity of the Aβ and the release of oxidative products in AD [89]. Other antiparkinson’s flavonoids, such as apigenin, were encapsulated in the phospholipid nanocarrier before nasal administration and proved to be delivered well within the brain, while upregulating dopamine releases in haloperidol-induced Parkinson’s models [90]. Naringenin nanoemulsion in capryol, tween 20, and water were also highlighted to mitigate amyloidogenesis in Aβ-induced SH-SY5Y by reducing the expression of APP, BACE, and phosphorylated tau in vitro [91]. Likewise, the nasal administration of co-loaded nanoemulsion of naringenin and Vit E reversed the MDA levels in 6-OHDA-induced PD rats [92]. A polymeric nanocarrier system using modified polycaprolactone was also used to encapsulate naringenin for the treatment of cerebral stroke. It was established that this nanoformulation can impede the release of pro-inflammatory factors and markers in deprived oxygen glucose-induced mesenchyme stem cells (MSC), which suggests their effectiveness in MSC-based ischemic treatment [93].

Some investigations have demonstrated the effects of encapsulating phenolic acids for neurodegenerative disease treatment. For example, by encapsulating gallic acid in chitosan nanoparticles, its circulation time after oral administration was prolonged. Additionally, it downregulated the secretion of pro-inflammatory factors, reduced infarction size, and alleviated neuronal impairments in ischemic rats [94].

For its part, resveratrol has been entrapped in lipid core nanoparticles to improve its therapeutic target delivery. The results established that the said formulation enhanced the neuroprotective activity in AD by attenuating the releases of neuroinflammatory markers and blockading kinases pathways (JNK, GSK-3β) [95,96]. Another type of encapsulation in PLA-PAS80 of resveratrol also showed promising neuroprotection effects in MPTP-induced mice [97]. However, Loureiro et al. optimized an ameliorated solid lipid nanocarriers functionalized with an anti-transferrin monoclonal antibody (OX26 mAB), which was defined as more efficient in BBB crossing and amyloidogenesis modulation [98]. Recent works have also evidenced the therapeutic enhancement of selenium nanoparticles loaded with resveratrol. These formulations not only reduced the Aβ aggregation in AD, but also impeded GSK-3β-mediated tau hyperphosphorylation and neuroinflammatory signaling pathways (STAT3, NF-kB, MAPK, and Akt) in AlCl_3_-AD rat models [99,100].

In 2014, an investigation on curcumin loaded in lipid-PEG conjugated with a monoclonal antibody in AD brain patients exhibited a high uptake of the said formulation and good clearance of Aβ aggregates [101]. Similar effects were demonstrated when conjugating curcumin-loaded polymeric nanocarrier (PLGA-CU) with glycopeptide (g7), brain targeting peptide (CRT), or serum albumin (BSA). It was discovered that these latter could enhance the BBB crossing and enhanced the phagocytosis of Aβ peptides via the modulation of macrophage polymerization [102,103,104]. On the other hand, solid lipid nanocarrier has shown better anti-amyloid and anti-inflammatory effects in AD models of 5XFAD mice, due to its better brain deliverance and glial astrocytes clearance [105]. Curcumin loaded in selenium nanoparticles covered with PLGA was also established to diminish extracellular Aβ peptides burden at the targeted AD lesion of transgenic mice (5XFAD) [106]. A co-loaded strategy of curcumin with piperine in a lipid nanocarrier built of glyceryl monooleate (GMO) and surfactants was also developed. This formulation has shown interesting attenuation of the α-syn fibrillation and toxicity induced by rotenone. Furthermore, it activated the anti-apoptotic of neuronal cells and the autophagy of fibril peptides, which ameliorates the neuronal abnormalities in PD models. The co-loaded drugs were stated to work synergistically once released to the PD target site [107]. The recent applications of phenolic-loaded nanocarriers in neurodegenerative diseases are synthesized in Table 1.

### 4.3. Polyphenolic Nano Delivery for Skin Inflammation and Wound

Most dermatological problems are issued by inflammation reactions. However, the inflammation of the skin represents chronic immune-inflammatory diseases involving psoriasis, dermatitis, and lupus erythematosus [108]. The psoriatic lesion is specified by epithelial hyperplasia, irregular differentiation of keratinocytes, infiltration of different inflammatory cells in the dermis, and vascularization [109]. The occurrence of these keratinocytes and immune cells enhances the release of pro-inflammatory factors around the lesions, which progressively amplify the psoriatic disease [110]. Atopic dermatitis, however, is associated with severe pruritus, persistent skin inflammation, and impaired skin barrier function [111]. Pathogenesis of this disease includes the upregulation of Th-2-driven inflammation, immunoglobulin E (IgE), and T-cells expressions [112].

The application of a nanodelivery system to ameliorate the efficacy of phenolic compounds has gained researchers’ attention during the last decade. Diverse strategies have been applied and achieved more advancement in the exploration of the enhanced method to deliver these kinds of compounds. With regards to EGCG, when entrapped in chitosan polymeric nanoparticles (CHI-EGCG-NPs), it has shown a four-fold effectivity in reducing inflammation responses and the proliferation of cultured keratinocytes. The topical application of this formulation on the imiquimod (IMQ)-induced skin model resulted in a significant decrease in immune cell penetration, as well as diverse psoriasis-related inflammatory markers [113]. Similarly, in dermatitis, the encapsulation of this catechin derivative in PEG-PLGA ameliorated the blockading of necroptosis (RIP 1, RIP 3, and MLKL expressions) in the 2,4 dinitrochlorobenzene (DNCB)-induced skin model, while the topical application resulted in noticeable downregulation of inflammatory cytokines releases. In the meantime, the synthesis of MAPK pathways (p-p38, ERK1, ERK2) in the epidermal layers of the dermatitis was also suppressed [114]. Co-loading of EGCG with Vit C in chitosan was established to quicken the wound healing of streptozocin-induced mice when administered intraperitoneally. The promotion of collagen deposition and angiogenesis, together with the blockage of immune cell infiltrations in the wounds, was speculated as the main synergistic effects of the co-delivered drugs [115]. A hydrogel nanocarrier entrapping EGCG was formulated for wound dressing. When applied to wounded rats, the hydrogel patch showed better wound healing efficacy than the commercial Neuskin^®^ tape, a mechanism which was determined as the regulation of inflammatory and growth factors, together with the promotion of collagen disposition [116].

A carbopol-based hydrogel nanocarrier incorporated with rutin nanocrystals was also developed to increase the efficiency of rutin in wound healing. The transdermal application of this preparation showed the protection of epidermal tissues induced by UV radiation [117]. An optimized ethosome formulation of rutin in lipid and ethanol has been also proven to protect against skin disorders, through their enhanced anti-inflammatory activity on keratinocytes cells and patient volunteers [118]. In the same way, optimal apigenin-loaded ethosome expressed a significant reduction of COX-2 levels in the UV-induced skin mice model [119]. Another work, conducted by Pleguezuelos et al., established a liposome formulation loaded with naringenin and tested for biocompatibility in 3T3 fibroblast cells. In vivo assessment demonstrated a better delivery of naringenin in the epidermis of TPA-induced skin mice, as well as great inflammatory-reducing effectivity [120]. A chitosan-coated liposome of naringenin was recently developed and proved to ameliorate the wound of experimental rats. The synergistic effects of chitosan and naringenin were stated as the principal therapeutical mechanisms of the optimized product in wound dressing [121].

Delivery trials of phenolic acids for skin inflammation have also been conducted. For illustration, the encapsulation of gallic acid in a polymeric nanocarrier composed of a lipid, glycerosome, and poloxamer has increased its deliverance in the skin. More importantly, the topical treatment of TPA-induced skin mice exhibited better anti-inflammatory effects, as explained by the impediment of leukocyte infiltration and activation [122]. Co-encapsulation of gallic acid with rutin in polymeric nanovesicle also exhibited positive effects in psoriasis treatment. It acts by blockading the hyperproliferation of keratinocytes, as well as protecting from psoriasis-related inflammation [123]. Recent works have established the effectiveness of hydrogel embodiment with gallic acid in wound healing. Topical application of such gallic acid nanovesicle speeds up wound regeneration and demonstrated to downregulate the expression of inflammatory cytokines [124,125]. Caffeic acid conjugated on nanofiber nanovesicle, formed with PCL and CH, ameliorated its delivery on fibroblast neonatal cell line (NHDF-neo) [126]. While conjugated on PLGA nanofiber, caffeic acid expressed better biocompatibility and wound dressing actions in the human fibroblast model [127].

Resveratrol was co-loaded with quercetin in lipid nanoparticles (oleic acid-S75) to improve their cellular intakes. When applied topically to a TPA-induced wound in mice, it rapidly regenerated the skin lesion by lowering the permeation of inflammatory cells [128]. An optimized co-loading of resveratrol and omega-3 (ω3) in lipid nanosystem has strengthened their anti-inflammatory potential in skin lesion diseases. The said formulation displayed longer circulation time and better inhibition of COX-2 and NO production in LPS-induced cells [129]. The incorporation of resveratrol in peptide hydrogel also boosted its wound-healing effectiveness by hindering the release of macrophage cytokines and promoting collagen restructuration [130]. Likewise, the hybrid composite encapsulation of curcumin by loading into polymeric (MPEG-PCL) or micelles (PEG-PCL, or PEG-PLA) vesicles before incorporation in hydrogel carriers (chitosan or dextran) resulted in sustained releases with a rapid wound dressing in vivo. Applications of these formulated hydrogels not only triggered the proliferation of fibroblasts, but also enhanced collagen agglomeration and angiogenesis in the tissue lesion [131,132,133]. This system also had positive therapeutic effects in the treatment of psoriasis, in which the pre-encapsulation in nanocarrier was supported to enhance the deliverance of curcumin to the targeted site [134]. Adhering curcumin on electrospun nanofibers was also developed for wound healing. Dai and coworkers developed curcumin loaded in gelatin nanofiber and discovered its advantages in inhibiting macrophages, as well as their cytokines releases [135]. Moreover, the encapsulation of silver nanoparticles functionalized with curcumin in chitosan was proven to be efficient in reducing wound lesions, without secondary irritation effects in the experimental model [136]. Lee and his team have developed a lipid nanocarrier co-loaded with epidermal growth factor and curcumin for treating diabetic chronic wounds. Their finding suggested that, in addition to the potential antioxidant of the formulation, it also helped in triggering the migration of fibroblasts and keratinocytes for wound closure [137]. Similar fibroblast migration was also determined when applying silica-based nanoparticles (CU-Si Np) loaded with curcumin on dermal fibroblast cells (HDF fibroblast) [138]. The detailed application of these different nanocarriers system loaded with phenolics and their mechanism of actions toward skin inflammation are summarized in Table 2.

### 4.4. Polyphenolic Nano Delivery in Inflammatory Bowel Disease

Inflammatory bowel disease (IBD) is a relapsing gastrointestinal tract (GIT) chronic inflammatory that comprehends ulcerative colitis and Crohn’s disease [139]. IBD is featured by an irregular immune response. Its pathogenesis is linked with the upregulation of NF-kβ pathways, along with the spectacular recruitment of epithelial and immune cells in the lamina propria [140]. Therefore, a rise in the transcription process increases the synthesis of inflammatory mediators involving interleukins (IL-1β, IL-6, IL-12, IL-23) and tumor necrosis factors (TNF-α). In IBD the highly expressed cytokine by T cells is the interferon-γ (IFNγ) that mainly contributes to the blood vessel’s vasculatures, apart from its immunomodulatory action [141]. However, the IL-13 cytokine, expressed by the natural killer cells (NK cells), is in charge of the epithelial barrier interruption [142]

In recent years, investigations on the delivery systems of phenolics for the treatment of inflammatory bowel diseases have emerged. An optimized polymeric nanosystem for the delivery of small interfering RNAs (siRNAs) complexed with EGCG was developed. The intrarectal administration of the obtained supramolecular showed significant decreases of TNF-α, prolyl hydroxylase 2 (PDH2), and inflammatory cell infiltration in the intestine tissue of a dextran sulfate sodium (DSS)-induced model [143]. In another study, ovalbumin was used to encapsulate EGCG, and it revealed its better cellular uptake. More importantly, the developed formula displayed noticeable suppression of pro-inflammatory markers expressions, such as TNF-α, IL-6, and IL-12, in vitro, while the intravenous injections ameliorated colitis in the DSS-induced model [144].

An advanced technique, by realizing pH response nanovector for delivering rutin to the inflamed intestine, was also established recently. It was constituted by polyglycerol as dendron and dodecyl sulfobetaine as surfactants. The brutal alteration of the environment from the gastric lumen to the mid-alkaline milieu of the intestine triggers the smooth release of rutin from the backbone shell [145]. Metallic-based nanoparticles coated with hyaluronic acid (HA) have also been established to load apigenin for targeting colitis. The oral gavage of API-Mn(II)@HA NPs displayed a better restoration of the damaged epithelial colon barrier, reduced inflammatory markers, and enhanced therapeutic effects against DSS-induced colitis mice [146].

Priyadarshi and coworkers, for their part, conjugated poly(amidoamine) dendrimers (PAMAM) with gallic acid before treating NIH 3T3 fibroblast cells. Firstly, a sustained release of gallic acid was remarked during the payload, appended with higher cellular uptake. In addition, the in vitro evaluations showed the downregulation of inflammatory markers NG-kB, IL-1β, and IL-6, when treated with the said nanocarrier [147]. Similar effects were found when using gallic acid as a stabilizer of tungsten-based polyxomerates nanocluster (W-POM NCs) in the DSS-induced colitis model [148]. Co-encapsulation of caffeic acid and the anti-inflammatory drug piceatannol in albumin resulted in a greater modulation of IBD-related inflammation. More specifically, a significant drop in HIF-1α levels and p65 was observed in colitis tissue [149].

The silk fibroin nanoparticle was thoroughly used to entrap the stilbene resveratrol for its delivery. In their work, Lozano et al. loaded resveratrol in cocoon source fibroin (RL-FNPs) by adsorption to apply for IBD. Their result emphasized that better anti-inflammatory impacts were found on TNBS model colitis treated with intracolonic injections of RL-FNPs than those treated with coarse resveratrol. The levels of chemokines such as CINC-1, MCP-1, and ICAM-1 dropped in the presence of RL-FNPS, while the peptide amounts in charge of repairing the epithelial barriers increased the mucins, TFF-3, and villin. A recent study used β-lactoglobulin for the encapsulation of this stilbene, which prolonged its release time, when taken orally, and showed greater clinical effects in the IBD mice model. Histological assessment supported that the BLG-RES acts in promoting the expressions of the anti-inflammatory cytokines IL-10 [150]. Polymeric nanocarrier, formed with PLGA, galactosamine, and tween 80, was also applied to enhance the oral delivery of resveratrol. This formulation not only increased the intestinal crossing of the loaded drug, but also displayed promising anti-inflammatory effects in RAW 264.7 macrophages [151].

**Figure 5:**
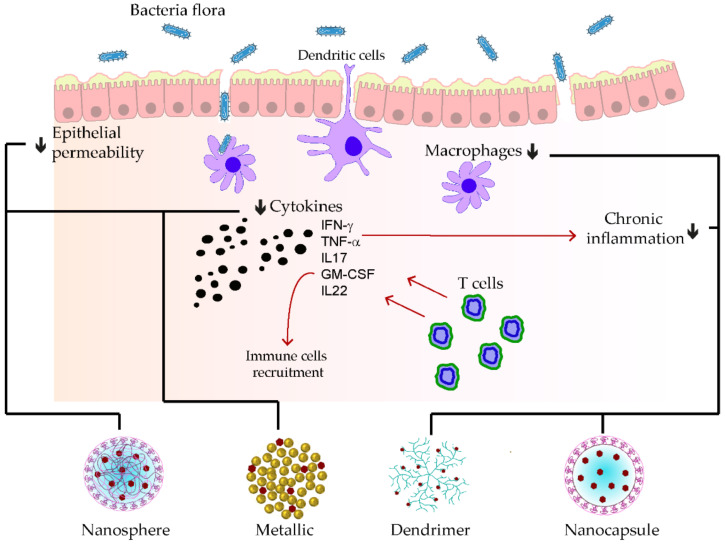
Examples of nanocarrier delivery of polyphenolics and their targets in inflammatory bowel disease. The black arrows up signify the increasing while the down ones indicate the decreasing effects. The red arrows stand for promotions. The black dots consist of inflammatory cytokines. The bold lines match the nanocarriers and their respective targets.

Homogenization within high pressure was utilized to stabilize the polymeric D-α-tocopherol PEG 1000 succinate curcumin (TPGS-stabilized curcumin) for its rectal delivery against ulcerative colitis. At first glance, the kinetic profile of the prepared nanoparticle was seven-fold superior to the free curcumin. Furthermore, tremendous anti-inflammatory effects were remarked during the reparation of colitis damages, which were explained by the synergistic effects of the TPGS and the nanosized nature of curcumin [152]. A pH-responsive CAP multilayer core-shell nanoparticle system (CAP_1_AG_4_CH_5_@CUNCs) to improve the delivery of curcumin nanocrystals was also developed for ulcerative colitis. The release of the curcumin nanoparticle was sustained, depending on the layer numbers and the colon pH surrounding 7.2. More than ten layers were necessary to ensure the optimal target delivery. Moreover, pronounced re-epithelization of mucosa leading to the hindrance of macrophage infiltrations was found after treating the colitis model with that of a multilayer curcumin-loaded core-shell nanoparticle [153]. In Figure 5, some examples of phenolic-loaded nanocarriers with their targets in the management of inflammatory bowel disease are illustrated.

### 4.5. Polyphenolic Nano Delivery for Metabolic Disorder

Metabolic disorders, mainly involving diabetes and obesity, are among the life-threatening illnesses of human beings nowadays [154,155]. Diabetes is manifested by the selective degradation of the insulin producer, pancreatic beta cells, or by insulin resistance or secretion deficiency. When these pancreatic beta cells are inflamed, inflammatory cells are recruited and infiltrate within the pancreatic islets. However, these cells produce inflammatory mediators involving the main cytokines IFNγ, TNF-α, and IL-1β, of which, overexpressions exacerbate the evolution of the pancreatic β-cells inflammation [156]. On the other hand, these pro-inflammatory cytokines also contribute to the reduction of insulin sensitivity [157]. Insulin resistance strongly impacts lipid metabolism and contributes to the onset of obesity, in general, along with adipose tissue hyperinflammation [158]. In obese people, the production of inflammatory mediators is promoted by resident and infiltrated immune cells in the adipose tissue through their MCP-1 stimulations [159]

The application of nanocarrier delivery has also received much interest in the management of metabolic diseases. As mentioned in Section 4.3, EGCG has been delivered to quicken the regeneration of impaired wounds in diabetic conditions [115,116]. Moreover, EGCG loaded in protein nanocarriers with a shell composed of β-lactoglobulin was developed to prevent metabolic syndrome. The administration of the formed nano-complex affects the levels of triglycerides and glycemia in high-fat-fed mice. The insulin sensitivity was also found to be ameliorated, which suggests its potential to reverse the severity of diabetes and obesity [160]. Similar therapeutic outcomes were determined while encapsulating EGCG in a hybrid formulation composed of PLGA in a hydrogel. This latter not only merely sustained EGCG releases, but also improved the regulations of LDL and HDL cholesterols in the HFD mice model [161].

Site-specific target delivery of rutin within a polymeric carrier composed of ethylene glycol-bis(succinic acid N-hydroxysuccinimide ester) tagged with argpyrimidine ligand (ARG-EG-RU) was also established and assessed with streptozotocin (STZ)-induced diabetic mice. The administration of ARG-EG-Ru considerably diminished the blood glucose levels and GHb in the STZ-induced models, while their insulin releases increased. A decrease in hyperlipidemia was also remarked after the treatment [162]. Amjadi et al. used a nanophytosomes system based on lecithin to entrap rutin. Their application in the STZ model resulted in a considerable alleviation of hyperglycemia and hyperlipidemia. In parallel, quick restorations of diabetic-damaged organs, such as the pancreas, liver, and kidney, were also discovered [163]. Co-application of rutin with selenium nanoparticles succeeded to improve diabetic nephropathy when administrated orally. It showed relevant cutback of inflammatory markers levels and JAK-2/STAT-3 signaling expressions. Adversely, the levels of SIRT-1 and Nrf-2 were raising, which suggested the renoprotective effects of this combination therapy [164]. With regards to apigenin, its delivery by encapsulation in polymeric nanocarrier (PEGlated-PLGA) for managing pancreatitis in a diabetic model has been promising. In addition to its ability to inhibit PSC growth by promoting apoptosis, the formulation also alleviated the overexpression of PSC-related inflammatory mRNAs (IL-6 mRNA, fibronectin mRNA, etc.) [165]. In a similar way, naringenin loaded in PLGA (N-PLGA) minimized the amount of glycated hemoglobin and triglyceride levels in STZ-induced diabetic mice after two doses of injections [166]. This compound has also been proven to be better delivered when encapsulated within nanoliposome in the treatment of non-alcoholic fatty acid disease (NAFLD), one of the obesity hallmarks [167].

Recent studies have synthesized phenolic acid delivered through nanocomposites for mitigating metabolic disorders. In 2018, Zhang et al. developed PEGlated hydroxyapatite functionalized with gallic acid and insulin for oral administration purposes. The said product presented a longer circulation time in the fluidic system and effective deliverance of insulin at the targeted site. It also lowered the glucose level of STZ-induced diabetic rats after 7 h ingestion [168]. Gelation of the gallic acid solution was made through convolution and supplemented with Konjac glucomannan. The composite hydrogel was further applied to diabetic wounds, which resulted in a considerable re-epithelization of the lesions and on-site collagen aggregation. In addition, an immunofluorescent study supported a diminution of inflammatory markers and COX-2 levels in the recovering injury [169]. A synthesized caffeic acid-loaded nanoliposomal vesicle formed of sodium deoxycholate (SDC) was developed for attenuating pancreatitis. In addition to its improved bioavailability and stability, its oral pretreatment counteracted the biochemical dysregulation, caused by L-ornithine in the pancreatitis model, through the modulation of the Nrf2 and NF-kB signaling pathways [170]. Phospholipid entrapment of caffeic acid (CA-PC) also exerted the amelioration of lipid serum profile of HFD rats, following 4 weeks of oral administration. The therapy not only reduced the amount of LDL-C and VLDL-C, but also rose the HDL level, which implies a favorable regulation of hyperlipidemia [171].

When entrapping resveratrol in PLGA through the emulsion method, beneficial therapeutic effects were evidenced, especially in alleviating hyperlipidemia. On oleic acid (OA)-induced Hep G2 cells, in vitro study showed that treatment with RSV-PLGA-NPs enhanced lipolysis of the high-fat cells, which resulted in the clearance of triglyceride accumulations [172]. Additionally, a PEGlated phenylalanine encapsulation of resveratrol (RES-PEG-PPhe) downregulated interestingly the level of blood glucose and iNOS overexpression in the STZ-induced diabetic rat model. It also triggered insulin production in the model [173]. Other delivery techniques based on lipid shell (dipalmitoylphosphatidylcholine and cholesterol) revealed a considerable increase in insulin degree and expression in STZ-induced β-T cells and further diminished the glucose levels in diabetic models. Similarly, the upregulation of SNARE proteins (Snap23, Stx4, and Vamp2) expressions in the insulin resistance of the STZ-induced model was demonstrated. The delivery system consisted of an oral administration of resveratrol encapsulated in lecithin-palm oil carrier [174]. The same diabetic model was used for the evaluation of gold nanocarrier efficiency in delivering resveratrol. Surprisingly, AuNPs ameliorated diabetic retinopathy by lowering the levels of adhered intracellular molecules (ICAM-1), TNF-α, and mRNA expressions and hindered the ERK1/2 signaling pathway [175]. The ligand coating of polymeric nanoparticles made of a lipid layer covered with DSPE-PEG5000-peptide (L-Rnano) was delivered properly to the targeted adipose stromal cells (ASCs) of the HFD rat model. The vein injection of the formulated products resulted in a successful diminution of fat and glucose level in the model, while the insulin expressions and obesity-related inflammation were regulated [176].

Regarding curcumin, its co-encapsulation with a coenzyme Q10 in PLGA improved its bioavailability. When administrating CoQ10, both diabetic inflammation and lipid metabolism were extenuated in STZ-induced models. Precisely, the synergistic effects of CoQ10 and curcumin in reducing CP, IL-6, and TNF-α, as well as the downregulation of cholesterols were determined [177]. Additionally, curcumin loaded in amphiphilic copolymer (PLGA-PEG-NPs), stabilized by CTAB, impaired the hepatic inflammation of diabetic rats. Orally administered CUR-PLGA-PEG-NPs displayed a lower regulation of COX-2, NF-kB, and TGF-β in STZ-induced diabetic serum, while promoting PPARγ expression [178]. Recent research performed a clinical trial delivery of this compound using an oral treatment of an optimized nanomicelle formulation for 12 weeks on patients with metabolic syndromes. The obtained result showed that the treatment has reduced noticeably the plasma triglyceride concentration and HOMA-b index. However, other metabolic syndromes indexes, such as anthropometric features, FBS, and HbA1c, have not been affected [179]. Table 3 synthesizes the current application of nanocarriers loaded with phenolic compounds and their actions towards their respective targets in metabolic disorders management.

### 4.6. Polyphenolic Nano Delivery for Cardiovascular Disease

Cardiovascular disease is one of the silent health burdens that enclose a cluster of disorders attaining the blood vessels and heart. Three main impairments cause this disease, involving atherosclerosis, arterial hypertension, and coronary artery diseases [181]. Inflammation plays a substantial role in the pathophysiology of each of these disorders. In atherosclerosis, the damaged arterial vasculature walls are the agglomeration points of lipid mediators. These lipids activate immune cells that trigger a continuous secretion of pro-inflammatory factors and adhesive molecules (VCAM and ICAM). These latter provoke the sustained recruitment of immune cells in the endothelium and enhance the formation of atherosclerotic plaques [182]. In coronary artery diseases, diverse mechanisms, such as immune complex-mediated and cell-mediated inflammation, contribute to its growth [181]. In addition to the depiction of high rates of inflammatory markers in patients with arterial hypertension, many investigations have also evidenced an abnormal ratio between MMPS and TIMPS [183].

To maintain the wellness of the cardiovascular system, the application of nanoscale delivery systems has recently gained the focus of researchers to improve polyphenolic bioavailability, as well as their therapeutic efficacy. In 2014, Hong et al. formulated a polymeric nanocarrier composed of aspartic acid and chitosan self-assemblage (EGCG-CS-PAA) for atherosclerosis therapy. The said formulation showed PH-responsive releases of EGCG, and its oral administration resulted in a considerable decrease of lipid deposition throughout the artery wall which was comparable to simvastatin [184]. Another work functionalized the core-shell of the nanocarrier with a CD36 ligand named (Palmitoyl)-2-(5-keto-6-octene-dioyl) phosphatidylcholine (KodiA-PC) to heighten the delivery and macrophage uptake of EGCG. Mouse vein injections for 22 weeks of this formulation affected the macrophage releases of MCP-1, TNF-α, and IL-6, while diminishing the aorta epithelial lesions [185]. Lipid nanoparticle coated with chitosan (CSNLCE) was also used and proven to ameliorate the delivery, together with the uptake of EGCG to THP1-macrophages and inhibit their cholesterol contents as well as the expression of MCP-1 [186].

With rutin, a silver nanoparticle system (rutin@AgNPs) was established to interrupt the thromboembolism in the vascular system. The release of rutin together with its physiological compatibilities improved considerably, while it prolonged the coagulation times (PT and aPTT). The thrombogenesis formation was also inhibited by injection of this nanoparticle at low doses in vivo [187]. Oral administration of nano-lipid vesicle of this biflavonoid also impacted thrombogenesis and clotting formations in the ferric chloride (FC)-induced microvascular model [188]. Nanolipid encapsulation of naringenin decorated with folic acid ligands (FA-LNPs/Nrg) expressed higher delivery and anti-atherosclerotic effects in the ApoE-/- model after oral treatment for 3 months. This therapeutic advantage was explained by the ease of FA-LNPs/Nrg to pass across different transmembranes, infiltrate the targeted atherosclerotic cells, and reduce atherosclerotic plaque burdens [189]. When co-encapsulating the lipid vesicle with indocyanine green (ICG) and decorated with VCAM-1 molecule to form a theranostic (V-Nar/ICG/LN), other beneficial information arose that supported the effectiveness of the nanoparticle to suppress mRNA expression of inflamed vasculature of different organs [189].

Phenolic acid-loaded nanocarrier systems also exhibited enhanced beneficial effects in CVD. He et al. developed gallic acid-loaded polymeric nanovesicles made with PTMC (poly(1,3trimethylene carbonate)) to impede the cytotoxicity of the clinically eluting stent drug used in severe atherosclerosis. Their finding supported that the formulated product improved vein endothelial cells (HUVEC) adhesions, while blockading artery smooth cells (HUASMC) development [190]. In addition, anti-platelet aggregation of gallic acid decorated dendrimer (GA-PAMAM) was also established [191].

To surmount the limitation of coarse resveratrol application in CVD treatment, Zou et al. synthesized a polymeric carrier prepared with PEG-L-Leucine for myocardial protection. This strategy prolonged the release time of resveratrol in the cardiomyocyte cells, along with satisfactory protection on ischemia reperfusion-injury in MI/RI model [192]. In the same manner, oral treatment of resveratrol loaded in PLGA prevented myocardial injury marker releases (troponin, LDH, AST), inhibited the expression of inflammatory cytokines, and upregulated eNOS expressions [193].

**Figure 6:**
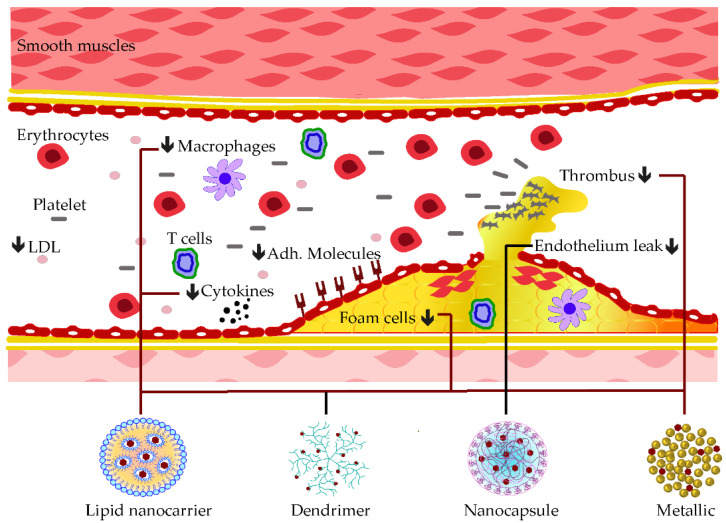
Examples of nanocarrier delivery of polyphenolics and their targets in Atherosclerosis. The black arrows down signify the decreasing effects. The black dots consist of inflammatory cytokines. The bold lines match the nanocarriers and their respective targets.

A mesoporous silica nanocarrier was established to embed curcumin compound before treating the myocardial defect induced by doxorubicin. In addition to the delivery improvement of curcumin, mesoporous nanoparticles also enhanced its anti-cardiotoxicity effects by reducing MDA levels and promoting the production of GSH, CAT, as well as SOD in cardiac tissue [194]. Synergistic anti-atherosclerotic effects were observed when co-encapsulating curcumin with atorvastatin calcium (Ato) in E-selectin-binding (Esb) ligand-coated liposome (T-AC-Lipo). The decoration with Esb ligands improved the cell delivery of the nanoparticle while curcumin and Ato worked interactively in reducing foam cell generation from monocytes and in blockading adhesions of molecules, such as ICAM-1 and E-selectin [195]. Some of these applications of phenolic-loaded nanocarriers and their respective targets for the mitigation of atherosclerosis are shown in Figure 6.

## 5. Conclusions and Future Perspectives

Many natural phenolic compounds have been proven to possess beneficial effects in the management of inflammation-mediated diseases, but their clinical applications are limited, due to their organism bioavailability and low target deliverance. Nanocarrier delivery systems have recently been widely used for improving the efficacy of diverse phenolic compounds. This strategy not only enhances the circulation time of the loaded phenolic in the body system, but also improves their deliverance to the expected site of actions. Our present review has synthesized the recent applications of nanocarriers loaded with common phenolics (EGCG, rutin, naringenin, apigenin, gallic acid, caffeic acid, resveratrol, and curcumin) for their effects in inflammatory-mediated diseases. Their physicochemical features, as well as their mode of action, have been discussed. Hybrid nanoparticle encapsulations were discovered to exhibit considerable advantages, due to their great variety of administration and high drug capacity loading. Furthermore, nanoparticle surface functionalization with biological molecules, such as peptides or/and nucleic acids, increased their effectiveness, in terms of target delivery and recognition. Co-delivery of different phenolics, or with approved drugs expressed as well better actions through their synergistic functions. However, side effects and nanotoxicology should be considered, as nanocarriers can cause allosteric sequestration on the cell membranes of vasculature, kidney, and liver. Sometimes, they may trigger immune defenses, which quicken their fast elimination by phagocytoses. Regarding the active targeting of functionalized nanocarriers, in some cases, knowing the specific receptors of the targeted cells remains challenging, which hampers the fast recognition and reachability of the formulated nanoparticles. So far, there is no explicit legislation for the standardization of medications formulated with nanocarriers, which holds their clinical extrapolation back. Consequently, given that the library of promising anti-inflammatory phenolic compounds has never ceased growing, future efforts should concentrate more on developing powerful and efficient strategies, while designing nanocarriers to specific target receptors through precision medicine. Harnessing the accrescent development of technologies, as well as the artificial intelligences in nanocarriers engineering and development (nanorobotics, pharmacytes, and simulations) also invigorate the advancement of nanomedicine. Finally, establishing standardized processing methods and preclinical application guidelines by the regulatory authorities helps in overcoming the clinical extrapolation barriers. Considering all this will give more allowance for the complexes’ inherent properties, modes of action, and the clinical translation.

## Figures and Tables

**Table 1:** Application of phenolic-loaded nanocarriers in neurodegenerative diseases.

Phenolic Compounds	Type of Nanocarriers (Size)	Targets	Mechanism of Action	Ref.
EGCG	Hybrid nanocarriers: EGCG@Se-Tet-1 coated	PC12 and NIH/3T3 cells	Disrupts Aβ aggregation and mitigates Aβ fibrillation. Protects PC12 cells from damages	[85]
EGCG	Polymeric nanocarriers: PEGylated PLGA (124 nm)	Alzeimer mice model (APP/PS1 mice)	Improves EGCG stability and efficacy by reducing neuroinflammation, Aβ plaque/peptide burden, and enhancing synaptogenesis	[82]
EGCG	Polymeric nanocarriers: PLA-PEG-PVA-EGCG (317 nm)	AlCl_3_-induced neurobehavioral deficit model	Attenuates neurobehavioral impairments, reduces the generation of Aβ plaque, neurofibrillary tangles, level of Aβ_1–42_, and Ache activity	[81]
EGCG	Micelle nanocarriers: PEG-PLGA-shRNA-RVG29 (152 nm)	BACE1, APP/PS1 mice	Reduces Aβ and BACE1 protein expression and enhances synaptogenesis, memory, and learning processes	[83]
EGCG	Selenium nanocarrier: Selenium (91 nm)	LPS-induced PC12 cells and SCI rats	Reduces the levels of inflammatory cytokines, improves locomotor capacity, and diminishes injury region	[86]
EGCG	Micelle nanocarriers –Hybrid (B6ME-NPs with SPIONS)	SH-SY5Y neuroblastoma cells	Improves the delivery to PD lesions and inhibits α-syn aggregation	[84]
Rutin	Phospholipid nanocarrier: RU-RPLC (<100 nm)	MCAO induced rats	Reduces toxicity and infiltrates well in BBB	[88]
Rutin	Metallic nanoparticle: Iron oxide-congo red-MNPS (oleic acid coated)	Aβ-induced SH-SY5Y cells	Inhibits Aβ-induced cytotoxicity and reduces the production of NO and ROS	[89]
Rutin	Solid lipid nanocarrier 100 nm	*Rattus norvegicus* rats	Infiltrates well in BBB	[87]
Apigenin	Lipid nanocarrier	Haloperidol-induced Parkinson’s model	Upregulates dopamine level	[90]
Naringenin	Nanoemulsion: Capryol 90-tween 20 (113 nm)	Aβ- induced SH-SY5Y cells	Decreases Aβ levels, APP, BACE, and tau expressions	[91]
Naringenin	Nanoemulsion: Capryol 90-tween 80-Vit E (38 nm)	6-OHDA-induced Parkinson’s disease in rats	Reverses PD symptoms in rats	[92]
Naringenin	Polymeric nanocarrier: PCL-gelatin-coated (192 nm)	OGD-induced mesenchymal stem cells	Reduces the levels of proinflammatory cytokines and biomarkers	[93]
Gallic acid	Lipid nanocarrier: GA-NPs	MCAO rats	Presents neuroprotection	[94]
Resveratrol	Lipid nanocarrier: RSV LNC (242 nm)	Aβ_1–42_-induced rats	Rescues deleterious effect of Aβ_1–42_	[96]
Resveratrol	Lipid core nanocapsule: RSV-LNC (242 nm)	Hypocamplal culture	Modulates neuroinflammation	[95]
Resveratrol	Polymeric nanocarrier: PLA-PS80	MPTP-induced C57BL/6 mice	Presents neuroprotection	[97]
Resveratrol	Solid lipid nanocarrier: RSV-SLN-OX26 (254 nm)	Endothelial human brain	Enhances Aβ-aggregation clearance, and blockades Aβ fibrillation	[98]
Resveratrol	Res-selenium-peptide: TGN-Res@SeNPs	AlCl3-AD model mice	Decreases Aβ aggregation in the hippocampus, and downregulates neuroinflammation	[99]
Resveratrol	RSV-Se nanoparticles (RSV-SeNPs)	AlCl3-AD rat model	Attenuates the mitochondrial dysfunction, downregulates inflammatory pathways: STAT3, GSK-3B, and triggers PI3K	[100]
Curcumin	Polymeric nanocarrier: PLGA-CU-g7 (204 nm)	Hippocampal cell cultures	Reduces the aggregation of Aβ	[102]
Curcumin	Solid lipid nanocarrier: SLNP-CU (Ni)	5XFAD mice	Improves neuroprotection and reduces astrocytic and microglia immune responses in AD-mice	[105]
Curcumin	Polymeric nanocarrier PLGA-NPs-CRT (128 nm)	(APP/PS1dE9) AD mouse model	Diminishes Aβ burden, astrogliosis, microgliosis, and memory impairment	[103]
Curcumin	Polymeric Se-NP-PLGA (160 nm)	(5XFAD) model	Diminishes Aβ burden and reduces memory impairment	[106]
Curcumin	Nanoliposomes Lip-PEG-CU-mAb (153 nm)	AD brain patients	Interrupts Aβ_1–42_ aggregations	[101]
Curcumin	Polymeric nanocarrier: Chit-BSA (143 nm)	hCMEC/D3 BBB cell model	Reduces NF-kB signaling and improves Aβ_42_ uptakes	[104]
Curcumin	Lipid nanocarrier: Cur-Pip-GMO-based NPs (93 nm)	Rat cell line, a cellular model of PD,	Inhibits α-syn aggregation and stimulates autophagy	[107]

**Table 2:** Application of phenolic-loaded nanocarriers in skin inflammation.

Phenolic Compounds	Type of Nanocarriers (Size)	Targets	Mechanism of Action	Ref.
EGCG	Polymeric nanocarrier: PEG-PLGA (176 nm)	DNCB-induced dermatitis model	Reduces ear and skin thickness, mitigates the inflammatory cytokines releases (TNF-α, IFN-γ, IL-4, IL-6, and IL-17A), blockades necroptosis (RIP1, RIP3, and MLKL expressions), and regulates MAPK pathways (p-p38, ERK1, and ERK2)	[114]
EGCG	Polymeric nanocarrier: CHI-EGCG-NPs (211 nm)	Cultured keratinocytes and IMQ-induced model	Reduces inflammatory responses and modulates psoriasis-related inflammatory cytokines	[113]
EGCG	Hydrogel nanocarrier: HG-Ag-EGCG (217 nm)	Subcutaneous wound in Wistar rats	Accelerates wound properties (modulates growth factors and cytokines)	[116]
EGCG	Polymeric nanocarrier: EGCG-Vit C-Gelatin, chitosan (200 nm)	Wound on STZ-induced diabetic mice	Promotes wound healing by raising collagen accumulation, promoting angiogenesis, and reducing inflammatory cell infiltrations	[115]
Rutin	Hydrogel nanocarrier: NC-RU-gel (447 nm)	UV-induced BALB/c mice	Increases the skin permeability of rutin	[117]
Rutin	Ethosomal nanocarrier: ETOH-PL90G-H_2_O (112 nm)	Keratinocyte cells (NCTC2544) and volunteer patients	Improves anti-inflammatory effect	[118]
Apigenin	Ethosomal nanocarrier (36 nm)	UV-induced skin inflammation	Reduces COX-2 levels	[119]
Naringenin	Liposome: NAR-Polysorbate 80-Lipoid^®^ (100 nm)	3T3 fibroblasts and TPA-induced mice	Reduces skin inflammation	[120]
Naringenin	Liposome: chitosan-coated naringenin nanoemulsion (105 nm)	Abrasion wound in rat model	Controls the delivery and ameliorates the wounds construction and skin regeneration	[121]
Gallic acid	Polymeric nanocarrier: Tween 80-chitosan (330 nm)	HaCaT cell line	Reduces keratinocyte proliferation and exerts protein protection in vitro.	[123]
Gallic acid	Hydrogel nanocarrier	Total skin defect model	Enhance wound healing by reducing the expression of IL-6, IL-1β, and TNF-α	[125]
Gallic acid	Hydrogel nanocarrier: GH/GGA	Skin wounded mice	Speeds up the wound healing by scavenging the ROS and promoting tissue regeneration	[124]
Gallic acid	Polymeric nanocarrier: liposome, glycosome GA polyxomer (70 nm)	TPA induced mice	Improves the skin target delivery and blockades leukocytes infiltration	[122]
Caffeic acid	Nanofiber nanocarrier: PLGA	In vitro scratch assay	Presents better wound healing properties on human fibroblast	[127]
Caffeic acid	Nanofiber nanocarrier: Chitosan-PCL/CCA	NHDF-neo cell line	Improves cell attachment	[126]
Resveratrol	Lipid nanocarrier: Oleic acid-S75 (79 nm)	TPA induced mice	Neutralizes the inflammatory response	[128]
Resveratrol	Lipid nanocarrier: DSPC/DOPE/ω3 (156 nm)	RAW 264.7 cell line	Inhibits COX and NO productions	[129]
Resveratrol	Peptide-hydrogel nanocarrier: Fmoc-FFGGRGD	Rat skin damage model	Inhibits macrophage production of pro-inflammatory cytokines	[130]
Curcumin	Hydrogel nanocarriers: PLGA NPS in hydrogel (150 nm)	IMQ-induced-C57/BL6 mice	Improves anti-psoriasis activity	[134]
Curcumin	Gelatin nanofiber mats	Rat wounded model	Improves the wounds by increasing fibroblast proliferation and migration, inhibiting macrophages, and reducing pro-inflammatory cytokines	[135]
Curcumin	Nanocrystal coated nanocarrier: Ch/CNC (Ag NPx/Cury)	Injured rat model	Accelerates wound closure and repairs tissues	[136]
Curcumin	Silica nanocarrier: CU-Si-Nps (36 nm)	HDF fibroblast cell	Enhances fibroblast migrations	[138]
Curcumin	Nanostructured lipid carrier: EGF–Cur-NLC (331 nm)	Punched wound on the skin	Accelerates wound closure	[137]
Curcumin	Hydrogel nanocarrier: PEG-PLA in dextran hydrogel (65 nm)	BALB/c mice	Accelerates angiogenesis, fibroblast accumulation, and wound healing	[133]
Curcumin	Hydrogel nanocarrier: MPEG-PCL in CCS-OA hydrogel (nano: 40 nm)	Injured tissue	Improves re-epithelialization of the injury	[131]
Curcumin	Hydrogel nanocarrier: PEG-PCL-PEG in hydrogel (micelle: 26 nm)	Wound model	Enhances cutaneous repair, and increases collagen content and wound maturity	[132]

**Table 3:** Application of phenolic-loaded nanocarriers in metabolic disorders.

Phenolic Compounds	Type of Nanocarriers (Size)	Targets	Mechanism of Action	Ref.
EGCG	Polymeric nanocarrier: EGCG-PLGA-in hydrogel (112 nm)	SC model	Increases delivery and reduces cholesterol, LDL-cholesterol while increasing HDL	[161]
EGCG	Hybrid nanocarrier HG-AG-EGCG	HFD-induced T2DM C57BL/6	Improves wound healing in diabetes by suppressing related inflammation	[116]
EGCG	Protein nanocarrier: EGCG-β-Lg (22 nm)	HFD obese mice	Lowers triglycerides amount in the model and improved glycemic homeostasis, as well as insulin sensitivity	[160]
Rutin	Lipid nanocarrier: Lecithin nanophytosome: (72 nm)	STZ-induced diabetic rats	Mitigates hyperglycemia and hyperlipidemia, reduces the induced damage of the kidney, liver, and pancreas in rats	[163]
Rutin	Polymeric nanocarrier: ARG-EG-RU (68 nm)	STZ-induced diabetic rats	Reduces glucose, GHb, and lipid levels, while increasing insulin amount	[162]
Rutin	Selenium nanocarrier: RU+Se-NPs	Sprague-Dawley rats	Upregulates SIRT-1, Nrf-2, and HO-1 and downregulates JAK-2/STAT3 pathways, as well as inflammatory markers	[164]
Apigenin	Polymeric nanocarrier: PEGlated-PLGA (160 nm)	Cholecystokinin- induced C57/BL6 mice	Inhibits PSC growth, promotes PSC apoptosis, reduce the expression of PSC-related inflammation	[165]
Naringenin	Lipid nanocarrier: NRG-Nano (98 nm)	Methionine choline-induced mice	Improved absorption and showed protection in fatty liver	[167]
Naringenin	Polymeric nanocarrier: N-PLGA (129 nm)	STZ-induced diabetic rat	Ameliorates diabetogenic (increases insulin level, and improves dyslipidemia)	[166]
Gallic acid	Hydrogel nanocarrier: GA-KGM	STZ-induced diabetic rats	Reduces the expression of IL-1β, TNF-α, and COX-2	[169]
Gallic acid	Polymeric nanocarrier: HAP-PEG-GA-INS (396 nm)	STZ-induced T1D rats	Reduces blood glucose level in diabetic rats due to a higher delivery of insulin	[168]
Caffeic acid	Lipid nanocarrier: CAPE-loaded-NL (309 nm)	L-ornithine induced rat	Modulated Nrf2 and NF-kB signaling	[170]
Caffeic acid	Lipid nanocarrier: CA–PC (168 nm)	HFD-induced hyperlipidemic model	Maintain hepatocyte structure which promotes lipid absorption	[171]
Resveratrol	Lipid nanocarrier: RSV-LPs (215 nm)	Glucose/STZ-induced β- TC cell	Reduces glucose and increased insulin level	[180]
Resveratrol	Metallic nanocarrier: RES-Au-NPs (20 nm)	STZ- induced diabetic rats	Overcomes blood-retinal barrier, reduces VEGF-1, TNF-α, MCP-1, ICAM-1, IL-6, and blockades of ERK1/2 signaling pathway	[175]
Resveratrol	Polymeric nanocarrier: RES/PEG-PPhe	Intestine of STZ-induced diabetic rats	Reduces glucose level and increases insulin level while alleviating intestine injury	[173]
Resveratrol	Solid lipid nanocarrier: SLN-RES (248 nm)	STZ-induced rats	Displays hypoglycemic activity, reduces Snap 23, Stx4 and Vamp2 in insulin resistance	[174]
Resveratrol	Polymeric nanocarrier: RSV-PLGA-NPs (176 nm)	OA-induced HepG2	Promotes lipolysis and mitigates hepatocellular proliferation	[172]
Resveratrol	Hybrid nanocarrier: L-Rnano (90 nm)	HFD-C57BL/6 J mice	Decreases fat mass and inflammation while improving glucose homeostasis	[176]
Curcumin	Polymeric nanocarrier: CU-PLA-PEG (117 nm)	STZ-induced rat	Reduces NF-κB activation, COX-2, TGF-β, and PPARγ expressions	[178]
Curcumin	Polymeric nanocarrier: CoQ10-PLGA (115 nm)	STZ-induced rat	Reduces CRP, IL-6, TNF-α, triglyceride, and total cholesterol levels in plasma.	[177]
Curcumin	Micelle nanocarrier: C3-CU-GRAS (12 nm)	MetS patients for 12 weeks	Reduces the level of triglyceride and HOMA-b index	[179]

## Data Availability

Not applicable.
